# Scaffolds for Wound Healing Applications

**DOI:** 10.3390/polym12092010

**Published:** 2020-09-03

**Authors:** Irina Negut, Gabriela Dorcioman, Valentina Grumezescu

**Affiliations:** Lasers Department, National Institute for Laser, Plasma and Radiation Physics, RO-077125 Magurele, Romania; negut.irina@inflpr.ro (I.N.); gabriela.dorcioman@inflpr.ro (G.D.)

**Keywords:** wound healing, scaffolds, natural polymers, synthetic polymers

## Abstract

In order to overcome the shortcomings related to unspecific and partially efficient conventional wound dressings, impressive efforts are oriented in the development and evaluation of new and effective platforms for wound healing applications. In situ formed wound dressings provide several advantages, including proper adaptability for wound bed microstructure and architecture, facile application, patient compliance and enhanced therapeutic effects. Natural or synthetic, composite or hybrid biomaterials represent suitable candidates for accelerated wound healing, by providing proper air and water vapor permeability, structure for macro- and microcirculation, support for cellular migration and proliferation, protection against microbial invasion and external contamination. Besides being the most promising choice for wound care applications, polymeric biomaterials (either from natural or synthetic sources) may exhibit intrinsic wound healing properties. Several nanotechnology-derived biomaterials proved great potential for wound healing applications, including micro- and nanoparticulate systems, fibrous scaffolds, and hydrogels. The present paper comprises the most recent data on modern and performant strategies for effective wound healing.

## 1. Introduction

Wound healing represents a highly complex physiological response of a living system to physical, chemical, mechanical or thermal injury [1]. It involves a cascade of cells, matrix components and other biological factors to act together in order to facilitate the healing and restore the tissue integrity. The wound healing process unites several overlapping phases: homeostasis, inflammation, proliferation/granulation, and remodeling/maturation. Still, when the healing course deviates from the normal path, the healing does not advance past the inflammatory phase. The deficiency of normal healing can be caused by burns, infections, or even complications arising out of pathological states [2].

To aid the wound care and the healing process, a suitable wound dressing must be applied at the wound site to protect the injury site from further external mechanical and microbial stress [3]. The customary wound dressings like cotton, bandages, and gauzes are dry, failing to provide a moist and active environment proper to the wound healing. Additionally, due to wound drainage, dressings tend to stick to the wound bed, and, when removed, they cause pain for patients [4]. To deal with these drawbacks, substantial efforts have been made to discover new protection that promotes wound healing and the repair of damaged tissue.

Since the first definition of the term “tissue engineering” in the late 1990s, biomaterial-based scaffolds have entered this domain in order to provide structural stability and suitable environment for cellular regeneration therefore “imitating” native tissue in functionality [5]. 3D scaffolds have been assessed for an extensive variety of applications ranging from bone [6], nerve [7], muscle, tendon/ligament [8] regeneration, and many more.

To produce these scaffolds, many synthetic and natural polymers have been examined. Appropriate selection of the material used to fabricate tissue-engineered scaffolds is a serious step concerning the success of the scaffold. Although natural polymers were among the earliest scaffold materials to be applied in clinical practice, due to their interactions with various cells and absence of an immune response, synthetic polymers were later applied due to their lower cost and a better functionality (regardless of the possible immune response to them or toxicity) [9]. Among synthetic polymers, poly(glycolic acid) (PGA) [10], polyethylene glycol (PEG) [11], poly(caprolactone) (PCL) [12], and poly(lactic-*co*-glycolic) acid (PLGA) [13] stood out for the creation of scaffolds. Synthetic polymers are also utilized in combination with natural ones, to improve/resolve concerns related to hydrophilicity, cell attachment, and biodegradability [9]. Various naturally derived polymers have been used as tissue scaffolds including collagen, chitosan, fibrin, gelatin, hyaluronic acid (HA), and silk. The use of synthetic and natural biomaterials generated scaffolds in the form of nanofibers, hydrogels, mats, 3D structures, sponges, foams, membranes, and nanogels [9]. Up to now, numerous strategies have been implemented to fabricate wound healing scaffolds, such as extrusion, molding, freeze-drying, rapid prototyping, and electrospinning. From these, electrospinning technique remains one of the most used, due to its simplicity, cost efficiency, flexibility, and scalability [14].

An ideal scaffold for wound healing applications should consist of these key features: fitting physical and mechanical properties, and excellent physiological background to enable cell adhesion, proliferation and/or differentiation. Furthermore, a scaffold should present a high porosity, a large surface area to volume ratio, an interlinked geometry, and to be flexible enough to accommodate the shape of the wound. Preferably, the scaffold must be biocompatible and biodegradable, with a degradation profile that is concomitant to the wound healing period of time. Another ideal characteristic of the scaffold must be to maintain a moist environment to provide essential cues to facilitate cellular adhesion, growth and migration, instigate angiogenesis, hurry granulation tissue formation, and facilitate re-epithelialization [15]. Moreover, scaffolds can be functionalized with many agents to enhance cellular responses and to boost the wound healing process.

In the following sections we will illustrate some aspects about wound healing phases, wound types and wound management. Additionally, we will talk about various natural and synthetic polymers, to produce scaffolds in the field of skin tissue regeneration, together with conventional and advances techniques for producing high-quality scaffolds. 

## 2. Wound Types and Wound Healing Processes

The largest organ in the human body is the skin. It is composed from three layers: the epidermis, dermis, and the fat layer, also known as hypodermis. The epidermis (the external layer of the skin) has the crucial role of maintaining the homeostasis of the body’s internal environment and at the same time protects the body from the external environment and from potential pathogenic bacteria. The inner layer, dermis, provides tensile strength for the skin through the support of the extracellular matrix (ECM). The dermis is where all blood vessels, nerves, hair follicles and sweat glands are situated. In its native state, skin is dry and acidic in nature (pH between 4 and 6.8) [16].

Destruction of the skin can weaken its function, which can be produced by a variety of means, including burns, trauma, surgeries, or lacerations. Consequently, correct wound care is imperative to avoid contaminations and additional complications, which may further result in impaired wound healing and in many morbidity and mortality cases.

**Figure 1:**
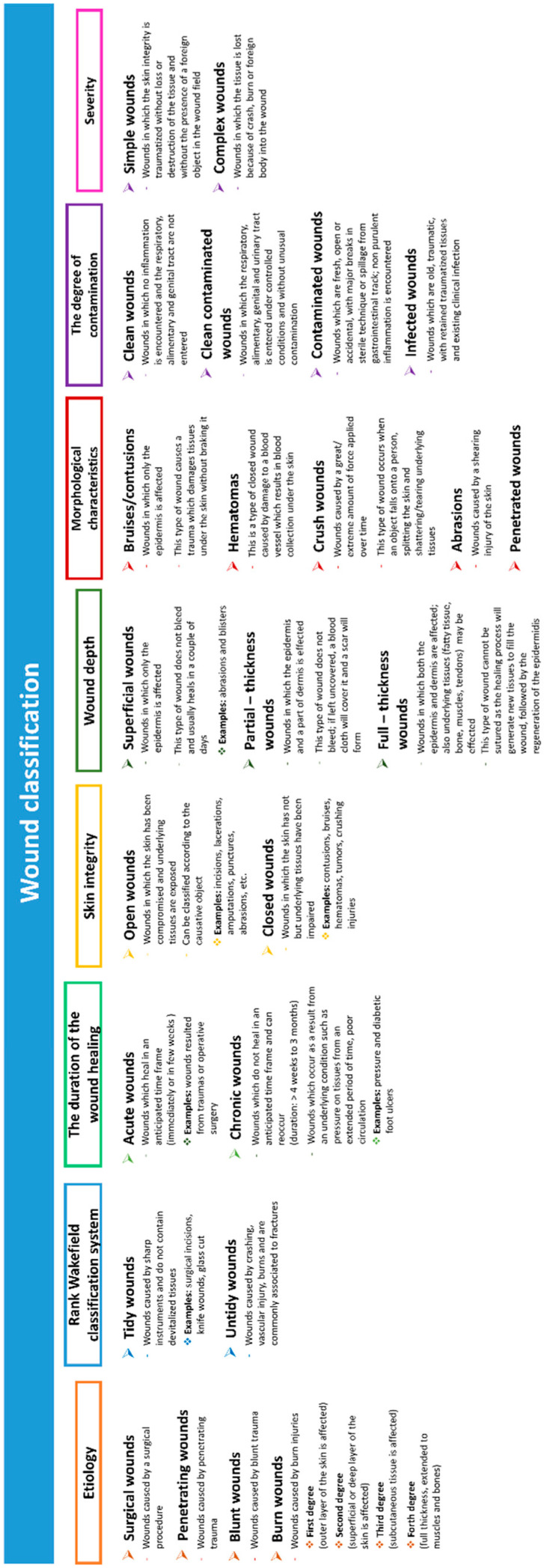
Wound classification.

When an individual has a severe injury to large areas of skin (such as burns), he is “open” to reduced local function, which can result in dehydration and infections and sometimes even death [17]. A wound can be described as a defect or a break in the skin caused by physical, chemical or thermal damage or as a consequence of a medical condition which would disturb the normal function of the skin [18]. The impairment of skin integrity may be caused by some systemic aspects, for instance the nutritional status of the individual, vascular and heart conditions, diabetes, or to extrinsic incidents (accidents, pressure, and surgical procedures). The skin impairment can range from simple breaks in the epithelium to deeper underlying such as tendons, muscles, even organs and bones [19]. An overview of wound types can be found in Figure 1. 

**Figure 2:**
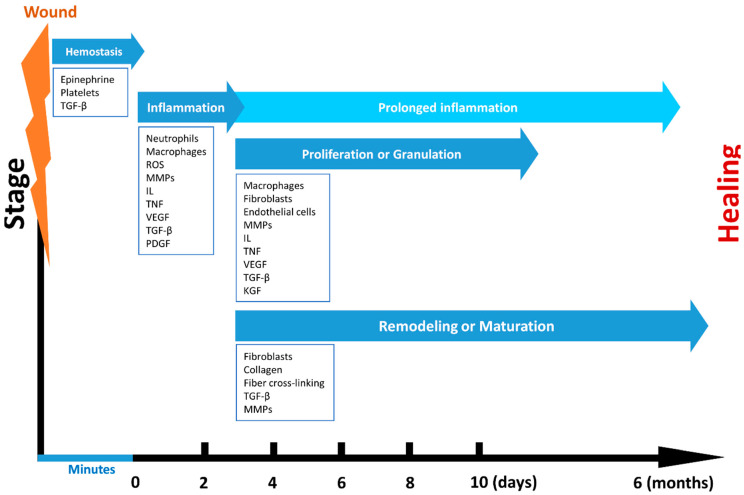
Wound healing phases.

An entirely healed wound can be defined as a wound which has returned to a normal anatomical structure, role, and appearance of the tissue, within a normal period of time [20]. Wounding, regardless of the cause and form, damages and disturbs the local environment. The wound healing is a multistep process which comprises complex biological sequences aiming to reestablish the skin function (Figure 2). Even though healing process is constant, it can be arbitrarily distributed into four main phases: (*i*) hemostasis, (*ii*) inflammation, (*iii*) proliferation or granulation, and (*iv*) remodeling or maturation. Still, skin regeneration and wound healing can be slow and can result in chronic inflammation, especially in patients with burn injuries [21].

The various stages of wound healing involve manifold cell populations and the action of mediators like growth factors (cytokines) (Table 1).

After the appearance of a wound injury, the first two stages, hemostasis and inflammation, start almost immediately. In these two phases, cytokines, growth factors, and reactive oxygen species (ROS) are formed in order to recruit cells to the wound’s site [20]. The interruption of the epidermal barrier takes place together with the discharge of the stored IL-1 and tumor necrosis factor-α (TNF-α) from keratinocytes. These factors signal adjacent cells to barricade the damage. The formed clot induces hemostasis and offers a ground for the invasion of inflammatory cells. The growth factors secreted by platelets, such as EGF, PDGF, and TGF-β, stimulate the enrolment of inflammatory leukocytes to the damaged site. Soon after, the contaminating bacteria are removed by neutrophils, which, in turn, are phagocytosed by engaged macrophages. The interactions at the wound site make monocytes undergo differentiation into macrophages. Macrophages cells have a central part in expanding the inflammatory reaction and also advancing the granulation tissue and release of different growth factors (EGF, TGF-β, and PDGF) [24]. Moreover, macrophages are stimulated by growth factors to produce matrix metalloproteinases (MMPs), reactive-oxygen species (ROS), and other growth factors such as PDGF, TGF-β, VEGF, and FGF [28].

The proliferative phase begins at about day 3 from the occurrence of injury and has a duration of 2–4 weeks. This stage is characterized by new tissue formation by endothelial, fibroblasts, and keratinocytes cells and the establishment of granulation tissue. The formation of granulation tissue starts at the wound’s site at ~4 days after damage [20]. Granulation is the effect of fibroblasts and macrophages providing a permanent source of VEGF and FGF essential to encourage angiogenesis and fibroplasias [29]. Fibroblasts are then phenotypically changed into myofibroblasts which themselves have effects on ECM borders in order to generate a constrictive force, enabling wound closure. The establishment of granulation tissue provides a sustenance matrix for epithelial cells to cover the wound surface in a process identified as reepithelialization. This stage generally implicates keratinocytes, which are major cell types found in the epidermis [30]. Additionally, blood vessels that are formed pass in the granulation tissue to allow blood flow and delivery of factors involved in wound healing [31].

The remodeling phase is the last of wound healing processes and mainly occurs from day 21 up to one year or even longer after the injury [20,29]. In the course of this phase, the formation of granulation tissue ends as fibroblasts either die by apoptosis or differentiate into myofibroblasts. This phase mainly implies the collagen refinement and its associated ECM. The resorption of the vascular capillary linkage marks the existence of mature scar tissue [32]. Under normal physiological conditions, the maturing scar presents balanced anabolic and catabolic processes that eventually will favor the maturation of the scar in a connective tissue. 

Even though processes involved in wound repair start almost immediately, not all wounds go through this cascade. For example skeletal tissue and eye go through different pathways [19]. In diabetic wound healing, inflammation can be prolonged, causing the wounds to become chronic.

Furthermore, there can appear modifications of tissues over time which can disturb the complete regeneration. Wound healing time can be diverse and some wounds may take >1 year to completely heal [19]. For example, systemic and local factors may interfere with the course of healing by causing instabilities in the repair processes, resulting in chronic, nonhealing wounds [19]. If the inflammation stage increases in time, it could lead to the dysfunctions of the early migratory effect and to the delay in healing. Besides, sustained chronic inflammatory reaction leads to ECM collapse and the establishment of necrotic sites. The failure to suitably change from the proliferation stage can lead to hypertrophic or excessive scarring [33].

## 3. Wound Management 

The wound management goal is to complete the healing in a rapid manner, with functional and esthetic outcomes [34].

For many years, wound management was based on covering the wound and the materials used for had a passive action in encouraging the healing process. However, in recent years, the wound management has been updated due to a greater understanding of the molecular and cellular processes involved in wound healing and preventing the wound from healing. Accordingly, the design and functionality of wound dressings has evolved in the direction of multi-functionality.

As mentioned above, wounds are unalike in terms of nature and features and are dependent on a diversity of factors such as the etiology, individual’s health condition, setting, and the manifestation of infections. Consequently, requirements of a wound dressing strongly depend on the type of wound. Nevertheless, some common features can be differentiated (Table 2).

Most of the mentioned characteristics depend on intrinsic features of the materials from which they are manufactured. The critical necessities of modern wound dressings include biocompatibility, no cytotoxic effect, no antigenic or inflammatory stimulation, a rate of biodegradability directly proportional with the rate of formation of new tissue, a release of incorporated bioactive constituents (drugs), and the control of possible infections [15,35].

Some of the key characteristics required for wound dressings/scaffolds, especially for burn wounds, are the water content and water-retaining properties. For these types of lesions, it is necessary to maintain the wound hydrated, to absorb exudates and to hurry the healing process by avoiding cellular dehydration and promoting collagen synthesis and angiogenesis [36]. An appropriate moisture control increases the healing rate, protects the wound from infection and reduces pain [37]. It is well known that exudates cause separation in the tissue layers of the wound, resulting in a slower healing. For that reason, exudates must be eliminated from the wound site with a dressing holding an appropriate drainage capacity.

However, it is essential for the wound to maintain some level of hydration, necessary for the healing process. The ability to preserve the humidity also prevents dressings to stick to the wound, therefore protecting the tissue from exposure and reducing the pain during the dressing exchange [38]. Dry wounds need dressings with a low vaporization rate, and exuding wounds the opposed. Therefore, the dressing needs to balance the absorption and release of liquids. Additionally, the dressings must allow the liberation of drugs and other bioactive compounds impregnated in the dressing material [39]. On the other hand, the complete limitation of fluid evaporation is to be avoided, as the fluid accumulation under the dressing may result in maceration and lastly in the establishment of infection. It has been found that the water vapor transmission rate (WVTR) from the skin is dependent on the wound type and healing stage. For example, for normal skin, WVTR is increasing from 204 g m^−2^ day^−1^ to 278 and for 1st-degree burns and granulating wounds is of 5138 ± 202 g m^−2^ day^−1^ [40]. A dressing is considered to be moisture retentive when its MVTR is <840 g/24 h/m^2^ [41]. High water vapor permeability increases the speed of dehydration at the wound site, therefore causing scar formation. In contrast, low water vapor permeability contributes to the deposition of exudates, delays wound healing process and consequently, predisposes the wound to infection [42]. Therefore, the chemo-physical (e.g., porosity, morphology) properties of the dressing must be adjusted to the wound type and to the grade of exudation.

The dressing/scaffold must maintain a normal tissue temperature which improves the blood flow to the wound site, encouraging epidermal migration [18]. The optimal cellular function is kept by maintaining a temperature comparable to that of the human body. Hypothermia results in vasoconstriction, further leading to a reduction of oxygen delivery to phagocytes, which diminishes the mitotic activity and growth factors liberation [43]. Oxygen is a critical element for wound healing. Under hypoxic conditions, wound healing is hindered as a result of reduced granulation and epithelization processes. Therefore, a desirable porosity and morphology of wound dressings and the ability to maintain moisture at the wound site are essential to accelerate and improve the wound healing [44].

A further required property is the ability to prevent colonizing infectious microorganisms or to fight against already installed ones. In healthy individuals, infection is avoided by triggering the immune system: macrophages cell induce the migration to the wound site and then perform phagocytosis of pathogens [45]. Nevertheless, if the immune system cannot eliminate pathogens, infection follows. This causes the deterioration of granulation tissue, growth factors, and ECM components (collagen, elastin, and fibrin), thus interfering with the normal wound healing process [45]. Therefore, the structure of desired dressings must offer a competent platform for drug delivery to the wound, controlling the release both spatially and temporally [46].

The technological factors are also needed to be taken into consideration, to ensure cost effectiveness of wound dressings and tolerate the change frequency. An adequate shelf life, suitable mechanical properties and the variation in degradation must match with the topical application and the timeline of healing processes.

All the above mentioned issues suggest the difficulty in finding a single ideal dressing, comprising all desirable features and capable of application to all wound types.

## 4. Current Polymeric Materials in Wound Healing 

When choosing a material to be used in wound treatment applications, it must possess particular properties. Of highest importance is the biocompatibility of the material. Is essential for the selected material to induce a proper response within the host organism. Up-to-date research recommended the integration of bioactive materials contrasting those that are inert, since bioactive materials usually interact with biological surroundings and impact the activities of cells [47]. 

Polymers are common used materials in wound dressing applications owing to their flexibility for chemical modifications, resulting in a chemical composition suitable for creating defined 3D structures and customized surface functionality [48]. Polymers are suitable as drug delivery systems as they have an unlimited variety in topology, dimensions, and chemistry. Taking into account the interest for wound management, polymers can be used as skin substitutes [15]. 

### 4.1. Natural Polymers

Natural polymers are mostly obtained from natural sources such as animal, microbial, and vegetable. In health-related applications, natural polymers have a number of advantages when compared with synthetic ones, such as biocompatibility, biodegradability, and/or biological activity. The origin of these materials make them suitable for the substitution of natural ECM structural components and skin cellular background [17]. Furthermore, natural-derived polymers share a chemical structure, rich in groups that can be adjusted with some derivatives, which results in the creation of versatile materials suitable for various tissue engineering requirements. Another key characteristic of natural biopolymers is that when subjected to enzymatic degradation, they produce by-products that are generally well tolerated by living organisms without triggering toxic reactions. Unfortunately, the high degradation rates/processes of natural polymers are quite difficult to control [26,49]. The most used natural polymers in wound healing applications are mentioned in Table 3.

### 4.2. Synthetic Polymers

When compared with natural polymers, synthetic polymers have the advantage to be synthesized and adjusted in a controlled mode to possess constant and homogenous physico-chemical properties as well as stability. Nevertheless, they are biologically inert; consequently they do not offer a therapeutic advantage as is seen in natural polymers [5]. They do not enclose impurities, are usually mechanically stable and they degrade in a controlled manner. However, they present an associated risk of toxicity. Widely used in wound healing applications, they can be categorized as hydrophobic and hydrophilic. Table 4 comprises synthetic polymers most commonly used in wound healing applications, as well as their properties. 

## 5. Engineered Scaffolds for Wound Healing

Even though wound dressings typically used in clinical practice (e.g., gauzes, absorbent cotton, bandages) are economical, they can provide a simple physical protection and are limited in terms of influencing/accelerating wound healing and preventing/treating infections.

The multidisciplinary association between tissue engineering and regenerative medicine includes aspects from engineering, biology, and material science and results in the progress of viable alternatives for organs and tissue regeneration/replacement. As an alternative treatment of wounds, tissue engineering has recently suggested a range of solutions, such as the introduction of scaffolds, to manage the treatment process.

Scaffolds, 3D structures, not only offer sustenance for tissue formation, but also can shield a wound, becoming an efficient “fence” against external contamination. It is of high interest that scaffolds need to provide physical support and interaction with cells to initiate physiological processes (cell adhesion, proliferation, and differentiation), leading to cell assembly into functioning units. Scaffolds should be both porous and biocompatible since cells need to adhere and travel through their networks. These characteristics must be considered when designing a scaffold for wound healing applications, while keeping its fabrication at high productivity with low costs [11].

Typically, a scaffold for wound healing can assist one of the following functions [61]: (i) It supports the delivery and retention of cells and different biochemical factors; (ii) It lets cells interact and connect by facilitating proper cell attachment and migration; (iii) It permits the flow of vital cell nutrients and released products; (iv) It modifies cells behavior by exerting mechanical and biological stimuli; (v) It mimics the ECM-like microenvironment in 3D space [62].

The fabrication and processing techniques have a high impact on the properties of resultant scaffolds. Ranjna C. Dutta et al. categorized these techniques into conventional and advanced (Table 5) [62].

Even though all fabrication methods are suitable, some matters associated to the scale-up, and cost attractiveness for realizing made-to-order scaffolds with distinct tissue specifications, remain to be addressed.

The ideal wound healing scaffold should provide: suitable physical and mechanical features to prevent infections and a physiological environment to facilitate cell adhesion, proliferation, and/or differentiation.

### 5.1. Physical and Mechanical Properties of Scaffolds for Wound Healing

The ability of tissue engineered scaffolds to absorb significant amount of fluid whilst maintaining a moist environment to the wound bed is crucial for wound healing and for skin regeneration. Generally, the fluid holding capacity of commonly used cotton pads is limited to ~6−7 times their dry weight. An excellent absorbent dressing/scaffold is compulsory for surgical wounds in order reduce pads replacement during the surgical intervention and for applications in chronic wounds which produce high amounts of exudates. To develop an ideal dressing envisioned for absorbing exudates, high absorbency characteristics (by means of channeled pores), satisfactory MVTR, and superior fluid retention/swelling are vital. For example, cinnamon (cin) was loaded into PCL/gelatin (PCL/Gel) nanofibrous for wound healing applications [42]. The results on water-uptake capacity of the obtained PCL/Gel scaffolds showed a capacity of 12.86  ±  0.32% and by adding cinnamon to the mat, the water-uptake capacity was increased. For PCL/Gel/1%cin, PCL/Gel/5%cin, and PCL/Gel/25%c, the water uptake capacity was of 13.63  ±  0.15%, 13.91  ±  0.5%, and 14.42  ±  0.6%, respectively. The study conducted by Shyna S. et al. involves the development of a blend from chitosan and PVA by a controlled freeze-drying process, thus giving it channeled pores [41]. Porosity of the scaffolds is critical for oxygen and nutrient transport into the wound site. It was emphasized that the best porosity for wound dressing is in the range of 60–90% [72]. The pore size on the top of the above mentioned dressing was of (17.25 ± 4.29) μm, and the bottom surface possessed pores with a size of (72.80 ± 17.93) μm. The MVTR of obtained dressings was of 200 g/m^2^/day for an 8-mm-thick dressing of 2:1 chitosan-PVA and was considered to be moisture retentive.

Besides taking up wound exudates, scaffolds must possess appropriate mechanical properties to accommodate different types of skin wounds. In the scientific literature, it was found that Young’s modulus of the skin varies from 10 kPa to 50 MPa, therefore, the scaffold must fit in this range [73]. The mechanical strength of scaffolds is considered to overcome the rapid degradation by enzymes and environmental changes. In this respect, Xin Zhao et al. developed electrospun 3D fibrous scaffolds for accelerated wound healing using a photocrosslinkable hydrogel based on gelatin methacryloyl (GelMA), that fit in above mentioned Young’s modulus range. GelMA scaffolds displayed typical stress-strain curves for viscoelastic materials owing to the gelatin segments having linear elastic behavior at low stress and non-linear region at higher stress [74]. M. Shu et al. reported the design and construction of physical polyelectrolyte complex hydrogel networks using chitosan and Heparin sodium (HS), aiming at achieving a wound scaffold (CS/HS) of high strength, toughness, and considerable flexibility [75]. CS/HS scaffolds showed superior strength and toughness (8.53 MPa and 11.32 kJ m^−2^), excellent extensibility (133.7%), and good self-recoverability. The integrity of dressings/scaffolds must be evaluated to prevent the contamination of the wound bed from dressing fragments. If the dressing is not very stable, it can lose its absorptive capacity during use and, hence, cannot be placed on wounded sites for longer periods of time [76]. Visual examination of freeze-dried chitosan PVA scaffolds showed that after conducting the dispersion test, all configurations of wound dressings did not display dispersion and maintained the integrity of the dressing, which ensured an easy removal after application [41].

Mechanical properties such as soft elasticity are of high importance for tissue engineered skin application, as elastic deformation of the scaffold material is known to regulate cell responses via biomechanical transduction [77]. Low fiber stiffness supports active recruitment of nearby fibers by cells, which facilitate cell migration [78]. In tissue engineering, studies have showed the status of matrix compliance in respect to cellular migratory capacities—soft 3D matrices stimulated a faster cell migration as paralleled to stiff ones. It is assumed that cells “sense” their way in a softer background which encourages cell migration [79].

### 5.2. Healing Properties

In addition to the importance of material selection, the inclusion of proper biochemical and/or biophysical stimuli could contribute to wound healing. Studies showed that local administration of therapeutic agents (such as antibiotics, antimicrobials, anti-inflammatory substances, cells, etc.) through the wound dressings/scaffolds can advance the wound healing process and can fight against wound infections.

As discussed in the introduction section, hemorrhage control is a crucial step for wound healing. Natural polymers (e.g., collagen, elastin) are particularly attractive wound dressing materials owing to their bioactivity, biocompatibility, and unique ability to bind specific growth factors and proteins, such as fibrinogen (Fb)—a plasma protein with a role in clot establishment through its activated form, fibrin. In this respect, Yuan et al. obtained by electrospinning a novel nanofibrous chitosan—Fb scaffold, proficient for PDGF sustained release, with submission to fibroblast migration and fast wound healing [80]. The authors showed that PDGF maintained functional activity throughout the electrospinning process. Still, the released PDGF was operative at encouraging fibroblast migration equivalent to a single 50 ng/mL dose of PDGF. A novel nanofiber composed from TEMPO-oxidized NFC and PEG, having different porosity (100 μm for 5% PEG and 200 μm for 10% PEG) was used to loading ZnO nanoparticles [81]. The obtained results displayed a reduced bleeding time in the case of the 5% PEG scaffold as compared to the 10% PEG one. In another study [82], an electrospun nanofibrous hybrid wound-dressing from PCL/collagen for sustained delivery of insulin into the wound bed was obtained. Insulin plays a crucial part in glucose uptake by skin cells and can stimulate keratinocytes and fibroblasts proliferation and migration. The structure of the wound healing scaffold demonstrated a slow release of insulin and enhanced mouse fibroblasts proliferation, as shown by the MTT assay.

Prevention of infection at the wound site is the primary goal of wound management strategies, which otherwise can delay normal healing progression. The use of polymeric fibrous scaffold constructs with encapsulated or attached antiinfectious components (such as antibiotics, nanoparticle, essential oils, antimicrobial peptides, etc.) has shown great potential as wound dressings to accelerate the wound healing process and to fight against wound infections. In this respect, A. Song et al. prepared three-dimensional synthetic cell-adhesive polypeptide hydrogel with antibacterial activity. The obtained data showed that higher weight ratios between the polypeptide and the PEG in the hydrogel composition were proper for cell adhesion and proliferation. Moreover, the obtained lysine gel displayed significant antibacterial activity against *E. coli* JM109 and *S. aureus* ATCC25923 microbial strains [83]. In a recent study, PCL-gelatin electrospun nanofibers, with average diameters of (725.943 ± 201.965) nm enriched with quercetin and ciprofloxacin hydrochloride (CH) were prepared. The in vitro antibacterial and antioxidant properties of scaffolds were evaluated by film-diffusion against *S. aureus* and DPPH assay; scaffolds had reduced the *S. aureus* growth on the agar plate and scavenged >50% DPPH (100 μM) in methanol [84]. Microfibrous constructs with Ag nanoparticles were obtained by single capillary electrospinning method. The in vitro diffusion study exhibited a controlled release of Ag nanoparticles from constructs. Similarly, the in vitro assessment of antibacterial activity of obtained scaffold demonstrated a high antibacterial activity and an inhibitory effect on the growth of both *S. aureus* and *E. coli* bacteria (antibacterial activity was dramatically increased, from 36.67 to 100%, after combining Ag nanoparticles with PCL, as compared to constructs without Ag) [85]. Biocompatible PCL/gelatin hybrid composite mats loaded with natural herbal extract from *Gymnema sylvestre* plant were prepared by Ramalingam et al. in order to prevent bacterial colonization [14]. The electrospun mats promoted cellular attachment, spreading and proliferation of human primary dermal fibroblasts and cultured keratinocytes, which are crucial parenchymal cell-types involved in the skin recovery process. Furthermore, the herbal extract loaded scaffolds possess all pre-requisite physical and biological properties to encourage the attachment, spreading, and proliferation of the fibroblasts and keratinocytes with potent antimicrobial behaviour against commensal pathogens such as *S. aureus* or *S. epidermidis*.

Excessive inflammation and reduced angiogenesis are two major difficulties for wound healing and skin regeneration. Therefore, treating the inflammation represents another crucial factor that needs to be taken into consideration. All through primary stages of inflammation, inflammatory cells secrete pro-inflammatory cytokines and boost phagocytosis to eliminate dead cells and injured tissue debris together with pathogens. Afterwards, anti-inflammatory cytokines are formed to diminish the inflammatory response and to stimulate tissue regeneration. As mentioned in the previous sections, macrophages have a crucial role in inflammation and immune responses. In this respect, Tanaka et al. developed uniform fibrin hydrogel scaffolds combined with SEW2871a monocyte/macrophage recruitment agent [86]. The culture conditions of the scaffold reduced the secretion of tumor necrosis factor-α (TNF-α)—a pro-inflammatory cytokine, and increased the secretion of interleukin-10—an anti-inflammatory cytokine, in mouse bone marrow-derived macrophages. The majority of cells that infiltrated the engineered scaffolds were macrophages expressing CD163, CD204, and CD206 anti-inflammatory macrophages markers, both in mice and human cells. In another study, Peng et al. reported on the fabrication of an advanced hydrogel from chemically modified HA, Dex, and β-cyclodextrin (β-CD) incorporating resveratrol and VEGF as anti-inflammatory and pro-angiogenic components for burn wounds. The obtained scaffold accelerated the splinted excisional burn wound healing, by obstructing inflammation response and encouraging microvascular formation while being biocompatible [87]. In another article, PU/SF (Antheraea mylitta) scaffolds were fabricated by blending and immobilization techniques. Dermal fibroblast NIH3T3 cells were seeded on EGF treated and untreated scaffolds. Fibroblast seeded PU/SF scaffolds were investigated for anti-inflammatory response in wound recovering potential, in comparison to the comercial Acticoat™ in third degree burn of streptozotocin induced diabetic rats. At 16th, 24th days, promising healing was achieved with faster granulation, enhanced collagenization, patterned re-epithelialization by EGF treated cellular immobilized PU/SF in normal, hyperglycemic burn. Inflammatory cytokines and immunoglobulins like IL-6, IL-8, IL-10 were examined, suggesting the anti-inflammatory role of PU/SF [88].

## 6. Conclusions and Future Prospects

The global wound care dressing market is expected to increase to $4.4 billion by 2019, from $3.1 billion in 2012, growing at an average annual rate of 5.7% [89]. As a consequence, the statistical data suggests that the wound care market forms a large segment of biomedical market.

Several aspects discussed in this paper concerning wound healing processes and the effects of exudate and mechanics on wounds could permit a profounder understanding of wound healing mechanisms. New techniques for the design and fabrication of wound dressings/scaffolds with advanced properties, suitable for keeping a moist environment while absorbing exudates, barrier against pathogens, drug delivery systems, healing agents, and matrixes for cells growth and anchoring, were briefly discussed.

Nowadays, there are still many problems encountered by healthcare professionals with the growing number of patients with microbial infections, wounds, and burns.

The next generation of wound healing scaffolds will involve the use of theranostic materials that will combine interactive and bioactive means together with therapeutic and diagnostic functionality into a single scaffold. It is envisioned that new technologies will integrate target biomarkers into scaffolds to monitor wound healing.

## Figures and Tables

**Table 1:** Cells and Growth Factors Involved in Wound Healing Phases.

Cells	Functions in Wound Healing	Ref.
Platelets	Thrombus formation Rich reservoirs of inflammatory mediators (including cytokines) Stimulus for inflammation	[22]
Neutrophils	First cells to infiltrate the injury site Phagocytosis and intracellular killing of invading bacteria	[23]
Macrophages	Clear debris and necrotic tissue, phagocytose and killing of invading bacteria Rich reservoirs of inflammatory mediators (including cytokines) Stimulate fibroblast division, collagen production and angiogenesis	[24]
Fibroblasts	Produce many components of the ECM (e.g., collagen, fibronectin, hyaluronic acid, etc.) Synthesize granulation tissue	[25]
**Growth factors**		
VEGF (Vascular endothelial growth factor)	Stimulates the angiogenesis in granulation tissue Stimulates endothelial cell proliferation	[26]
FGFS (Fibroblast growth factors)	Proliferation of fibroblasts and epithelial cells, matrix deposition, wound contraction, angiogenesis Accelerates the formation of granulation tissue	[27]
KGFS (Keratinocyte growth factors)	Proliferation and migration of keratinocytes	[28]
PDGF (Platelet-derived growth factor)	Mitogenic for both endothelial cells and fibroblasts Chemoattractant for neutrophils and fibroblasts Fibroblast proliferation and collagen metabolism The only growth factor currently approved by the FDA	[24]
EGF (Epidermal growth factor)	Differentiation, proliferation, migration and adhesion of keratinocytes Formation of granulation tissue	[24]
IL-1 (Interleukin-1)	Neutrophil chemotaxis Fibroblast proliferation	[24]
TGF-Β (Transforming growth factor-β)	Mitogenic for fibroblasts Chemotactic for macrophages Indirect stimulation of angiogenesis and collagen metabolism	[24]

**Table 2:** Features of Wound Dressings.

Physical	Chemical	Technological
Maintain proper moisture at the wound bed Permeable to water and gas Ability to absorb exudates and blood at wound site Mechanical protection Adaptability to wound type and body shape Protecting/acting against infectious agents Maintaining temperature To be easily applied and removed with minimal frequency	Biocompatible Suitable degradation rate Non-toxic Non-inflammatory Non-allergenic Antimicrobial action	Economical process of preparation High reproducibility Mechanical stability Easy sterilization Long shelf life

**Table 3:** Natural Polymers.

Polymer	Advantages	Disadvantages	Role in Wound Healing	Ref.
Alginate	Biocompatible Resistance in acidic media Biodegradable Relative low cost Low toxicity Gelling properties	Overstimulation of fibroblasts	Ability to absorb fluids Maintains wound moist Promotes granulation tissue formation Stimulates monocytes to produce elevated levels of cytokines	[50,51]
Collagen	Major protein component of the ECM Good biocompatibility High mechanical strength Good cell recognition	Contamination by viral Difficult to process High cost when resulting from recombinant technologies	Indispensable structural supportive role in connective tissue Cell adhesion properties as it binds with extracellular integrin receptors through arginine/glycine/aspartate binding sites	[52,53]
Chitosan/ Chitin	Biocompatible Biodegradable Nonantigenic Nontoxic Oxygen permeability	Poor stability	Improves fibroblasts, macrophages, and inflammatory cells functions Antimicrobial activity Rapid bone regeneration at initial stages Enhances the granulation of wound Its degradation products take part in the makeup the ECM and cartilage	[49,50,54]
Cellulose	Biocompatible The most abundant molecules in nature Plant origin cellulose can be isolated with cheap procedures	Expensive purifying processes	Stimulates PDGF, FGF and EGF, which increase granulation tissue formation and vascularization	[50,55]
Dextran (Dex)	Biocompatible Colloidal Hydrophilic Inert in biological systems Hardly affects the cell viability.	Limited solubility	Accelerates polymerization of fibrin in vivo Stimulates the structure of the fibrin clot Stimulates macrophages	[56,57]
Fibrin/fibroin	High mechanical strength excellent Biocompatibility Minimal adverse effects on the immune system	Inflammation Degradation	Clot formation Contributes to bleeding stopping The basis for cells migration during wound healing Substrate for platelets, endothelial cells, fibroblasts, and macrophages	[50]
Gelatin	Denatured form of collagen Excellent biodegradability Non-antigenicity Cost efficiency	It dissolves as a colloidal sol at or above 37 °C, and gels near room temperature Gelatin is frequently cross-linked/combined with other polymers	Facilitates cell adhesion and proliferation	[58]
HA	Forms a smaller part of the ECM Biocompatible Soluble in water Nonallergenic	Rapid enzymatic degradation in physiological media	Stimulates fibroblast proliferation and collagen deposition Enhances keratinocyte proliferation	[59,60]

**Table 4:** Properties of Commonly Used Synthetic Polymers for Wound Healing.

**Polyvinyl alcohol** **(PVA)**	Biocompatible Nontoxic Hydrophilic Water soluble pH sensitive
**PLA**	Biocompatible Biodegradable The degradation products are absorbed by the body through natural metabolic pathways Nontoxic Hydrophobic Structurally stable
**PGA**	Biocompatible Biodegradable (by hydrolysis; it produces CO_2_ and lowers the local pH leading to cell and tissue necrosis. More hydrophilic than PLA High tensile strength
**Polyurethane** **(PU)**	Biocompatible Degradation rate can be adapted Potential side effects of degradation products Tough and durable
**PLGA**	Biocompatible Biodegradable Degradation rate can be controlled by adjusting monomer ratios
**PCL**	Biocompatible Biodegradable Slower degradation rate than other polyesters Hydrophobic Semicrystalline Good elastic properties
**PEG**	Biocompatible Nonbiodegradable Bioinert Hydrophilic Resistant to protein adsorption
**Polydimethylsiloxane** **(PDMS)**	Bioinert Nonbiodegradable Compatible with blood Low toxicity Hydrophobic surface Antiadhesive properties Exceptional elasticity when lightly cross-linked Good thermal stability
**Polyethylen** **oxide** **(PEO)**	Non-toxic Biocompatible Non-immunogenic Hydrophilic Flexible
**Polyvinyl pyrrolidone** **(PVP)**	Biocompatible Biodegradable Environmental stability Low cytotoxicity High chemical and thermal resistance Affinity to complex hydrophilic and hydrophobic substances Very good solubility in water and organic solvents

**Table 5:** The Most Commonly Used Methods for the Fabrication of Scaffolds.

Conventional	Advantages	Disadvantages	Suitable Materials	Ref.
Solvent casting/particulate leaching	Control over porosity and crystallinity Minimal material needed	Residual solvents and limited mechanical features Pores not interconnected	PU PCL PLA	[63,64]
Extrusion	Control over porosity Obtaining 3D scaffolds	It uses pastes/pallets and a volatile solvent High temperatures or pressure needed	PCL PLA Calcium phosphates	[65,66]
Molding	Control over porosity	High temperature for nonamorphous polymer	HA PLGA Gelatin	[64]
Freeze-drying	High temperature and separate leaching phase not mandatory	Lower porosity than in other methods	PLGA PGA	[63]
Gas foaming	Free of harsh organic solvents and control over porosity	Limited mechanical features and poor pore interconnectivity	PLGA	[63]
Supercritical fluid processing	Controllable cellular structure and exemption of toxic organic solvents The blowing agent is non-toxic, non-flammable, chemical stable Its low critical temperature can protect heat-labile compounds from thermal degradation	Time consuming	PVA PEG	[67,68]
Fiber bonding	High surface to volume ratio and high porosity	Limited application to some polymers	PGA	[63]
Fused deposition modeling	Control over pore size, morphology, and interconnectivity Can have a linear print speed of 10–50 mm/s	Temperatures used are generally too high for the inclusion of cells or bioactive molecules	PCL TCP	[69]
**ADVANCED**				
Rapid prototyping - 3D plotting - 3D printing - Stereolithography -Selective laser sintering	Excellent control over geometry and porosity	Limited application to some polymers and the use of expensive equipment	PCL PLGA PVA PEEK PDLLA	[63]
Electrospinning	Control over porosity and crystallinity; diameter and orientation of fibers can be manipulated	Pore size decrease with fiber thickness	PCL Collagen PLA EVOH Chitin	[63]
In situ photopolymerization	Spatial and temporal control over polymerization, fast curing rates (< a second - few minutes) at room or physiological temperatures, and minimal heat production hydrogels can be created in situ from aqueous precursors To form complex shapes	Biological sensible molecules can degrade Limited material choice Limited mechanical properties of scaffolds	Polyurethane PEGMA PHBV Polyacrylamide	[64,70]
High internal phase emulsion (HIPE)	Good pore morphology	High processing temperatures Limited polymers	Biodegradable polymers	[64]
Self-assembling peptides	Compatible with in vitro cultures Peptides can be assembled into complex architectures (fibers, sheets, spheres)	The use of expensive materials The scaffold size is limited	RAD16-II RAD16-I EAK16-II KLD12 P11-4 MAX1	[64,71]
